# Control and Resolution Mechanisms of the Inflammatory Response

**DOI:** 10.1155/2014/387567

**Published:** 2014-12-01

**Authors:** Víctor M. Baizabal-Aguirre, Carlos Rosales, Constantino López-Macías, Marisa I. Gómez

**Affiliations:** ^1^Centro Multidisciplinario de Estudios en Biotecnología, Facultad de Medicina Veterinaria y Zootecnia, Universidad Michoacana de San Nicolás de Hidalgo, Km. 9.5 Carretera Morelia-Zinapécuaro S/N, 58893 Morelia, MICH, Mexico; ^2^Departamento de Inmunología, Instituto de Investigaciones Biomédicas, Universidad Nacional Autónoma de México, Apdo. Postal 70228, Ciudad Universitaria, 04510 México, DF, Mexico; ^3^Unidad de Investigación Médica en Inmunoquímica, Hospital de Especialidades del Centro Médico Nacional Siglo XXI, Instituto Mexicano del Seguro Social, Avenida Cuauhtémoc 330 Col. Doctores, 06170 México, DF, Mexico; ^4^Nuffield Department of Medicine, Peter Medawar Building for Pathogen Research, University of Oxford, South Parks Road, Oxford OX1 3SY, UK; ^5^Instituto de Investigaciones en Microbiología y Parasitología Médica (IMPaM), Universidad de Buenos Aires, Consejo Nacional para la Promoción de la Ciencia y la Tecnología, Paraguay 2155 Piso 12, 1121 Ciudad Autónoma de Buenos Aires, Argentina

Inflammation is beneficial to the organism because it represents one of the first barriers against external and internal stimuli. It is a complex process in which a number of cells and molecules play different roles in a coordinated and well-controlled manner. However, a failure of the mechanism that self-regulates and resolves the process may lead to chronic inflammation, and this in turn may cause degenerative diseases such as cancer, diabetes, and autoimmune and cardiovascular diseases. Therefore, a deep knowledge about the cellular and molecular mechanisms used to resolve inflammation is mandatory to design tools and strategies to control it. This task is not easy taking into account that different signaling pathways activate several molecules involved in the resolution of the inflammatory response and some of them interfere with cellular activities unrelated to the resolution phenomenon. In addition, many reports have shown that several molecules activate or inhibit inflammation depending on the tissue or the physiological context. Furthermore, it has been observed that inhibition of several molecules considered as proinflammatory has resulted in the intensification of the inflammatory response. Having this is mind, this special issue has gathered original and review articles that will help us to expand our knowledge on the complex process of the inflammation control and resolution. More importantly, the papers presented in this special issue are a good reference to recognize what type of studies is missing and the way we could fill the gaps.

As stated before, the inflammatory response is a complex molecular process triggered not only by chemical structures derived from microorganisms but also by structures delivered when cells suffer a nongenetically programmed form of death such as that caused by chemotherapeutic drugs, necrosis, necroptosis, and pyroptosis. The review article by Sangiuliano et al. explains the important role that the redox microenvironment (ROS and RNS) plays to control and resolve inflammation caused by molecules produced during cell death. These authors also point out how the oxidation of several thiol-containing proteins acts as a regulatory molecular switch to reverse the effects of proinflammatory cytokines. Resolution of inflammation, following apoptotic cell death, is also fundamental to keep homeostatic conditions. In an interesting paper, Byun et al. propose the experimentally tested hypothesis that apoptotic cells induce the expression of the hepatocyte growth factor (HGF) through activation of the cyclooxygenase-2 (COX-2)/prostaglandin E_2_ (PGE_2_) signaling in macrophages. The results presented indicate that the HGF/c-Met and COX-2/PGE_2_ signaling pathways communicate with each other via a positive feedback cross-talk loop, enhancing the synthesis of PGE_2_ and HGF, that in turn act as anti-inflammatory and antifibrotic molecules during the interaction of apoptotic cells with macrophages.

Taking into consideration that resolution of inflammation is a complex phenomenon that involves the activation or inhibition of many molecules, Alves-Perez et al. discuss in this issue recent reports about key signaling molecules (i.e., CDK, ERK1/2, p38, JNK, cAMP, PI3K, and NF-*κ*B) which proved to be important for the inflammatory response resolution and leukocyte survival (annexin A1, hydrogen peroxide, and TRAIL), and how their regulation induces the tissue to recover its homeostatic state. Besides, the authors argue that more* in vivo *studies are certainly necessary to use these molecules as effective therapeutic targets in chronic inflammatory diseases.

It is well known that obesity is a public health problem in developed and developing countries because complications such as metabolic syndrome have become one of the main causes of death. The comprehensive and well-documented review by Khan et al. is a good reference to understand the complex relations between obesity, metabolic syndrome, and chronic inflammation. Importantly, as the authors' state, this chronic inflammatory response may lead to different types of cancers. According to the authors' proposal, one of the key strategies to resolve obesity-induced chronic inflammation is the ingestion of the anti-inflammatory omega-3 fatty acids, among other strategies.

A significant body of literature has emerged during the last fifteen years describing the signaling cascades induced by Toll-like and Nod-like receptors in response to microbial PAMPs. In this issue, Oviedo-Boyso et al. have extensively reviewed these signaling cascades with a main focus in the interconnections among pathways triggered by TLRs and NLRs that serve to initiate host inflammatory responses critical for pathogen eradication during disease. Although the synergistic activity of these receptors has been proved, the authors emphasize the need for a better understanding of which molecules are involved in such synergistic effects. Moreover, novel signaling pathways that regulate the induction of inflammatory mediators in response to invading microorganisms are being permanently described. In this issue Silva-García et al. have revised recent findings about the role of Wnt/*β*-catenin signaling in the control of inflammatory responses induced by pathogenic bacteria. Current and future studies including different pathogenic microorganisms and cell types will allow the identification of key interconnection points of several signaling pathways that may be used as novel targets to control inflammation during infections and noninfection diseases.

The role of innate immunity in shaping adaptive immune responses as well as the capacity of T cell subtypes to modulate inflammation is actually well-recognized. CD4^+^CD25^+^ T cells (Tregs) in particular have acquired especial attention due to their potential to modulate several inflammatory cascades. In this issue Zhang et al. have described the role of Tregs in the attenuation of fine particle matter-induced inflammation in endothelial cells. Their findings significantly contribute to elucidating signaling cascades that could serve as potential targets to treat the adverse effects of fine particle matter in cardiovascular diseases.

An important aspect of immunity is the intimate relationship between innate and adaptive responses to control inflammation. Allergy responses have always been considered as an aberrant immune-mediated inflammatory reaction against harmless substances. In particular, due to its early introduction in a person's life, allergy to cow's milk proteins is one of the earliest food allergies. In this issue Jo et al. discuss the role of cellular immunity in allergic inflammation and tolerance induction against cow's milk proteins. The role of innate dendritic cells in initiating an immune response against milk proteins via activation of T cells is presented. Then, the interplay between Th2 cells and IgE-producing B cells and at the same time the downregulation of regulatory T cells (Treg) leading to cow's milk allergy are described. The cellular mechanisms discussed may help elucidate how to better control this one and other types of allergies.

Hepatitis C virus (HCV), Epstein Barr virus (EBV), human papillomavirus (HPV), and human T-cell lymphotropic virus type-1 (HTLV-1) are considered important risk factors for the induction of tumour malignancies. Inflammatory response elicited by these viruses could lead to the eradication of the infection; however, it could also promote tumour development. Although the mechanisms involved in this apparent paradox have been intensively investigated, there is little known about how the cells participating in the control and resolution of inflammatory responses could contribute to the carcinogenic process. The review article by Ouaguia et al. presents the current evidence on the natural and induced Tregs contribution to the generation of HCV-, HTLV-1-, and EBV-associated cancers through the promotion of the control or resolution of the inflammatory response triggered by these viral infections.

*Víctor M. Baizabal-Aguirre* *Víctor M. Baizabal-Aguirre* *Carlos Rosales* *Carlos Rosales* *Constantino López-Macías* *Constantino López-Macías* *Marisa I. Gómez* *Marisa I. Gómez*

